# Telehealth in palliative care is being described but not evaluated: a systematic review

**DOI:** 10.1186/s12904-019-0495-5

**Published:** 2019-12-13

**Authors:** Sophie Hancock, Nancy Preston, Helen Jones, Amy Gadoud

**Affiliations:** 1 International Observatory on End of Life Care, Furness Building, Lancaster University, Lancaster, LA1 4YG UK; 2 St Catherine’s Hospice, Lostock Hall, Lostock Lane, Preston, PR5 5XU UK; 3 Trinity Hospice, Low Moor Road, Bispham, Blackpool, FY2 OGB UK

**Keywords:** Telemedicine, Palliative care, Systematic Review

## Abstract

**Background:**

Telehealth is growing and its application in palliative care is seen as a solution to pressures on palliative care services. A 2010 UK review reported growing awareness of telehealth in palliative care but a lack of evidence-based research to support its use. The primary aim of this review was to describe the current use of telehealth in palliative care in the UK and evaluate telehealth initiatives against a digital service standard. The secondary aim was to explore whether telehealth results in a reduction in emergency care access.

**Methods:**

Systematic review of the literature with thematic synthesis. Records were screened and data extracted by two reviewers. EMBASE, MEDLINE, CINAHL, Psychinfo and Cochrane central register for controlled trials were searched using pre-defined terms. Hand searching of conference literature, thesis databases and citation tracking was also conducted. The protocol for this systematic review was registered with PROSPERO and can be found at http://www.crd.york.ac.uk/PROSPERO/display_record.php?ID=CRD42017080038.

**Results:**

The search identified 3807 titles and 30 studies were included in the review. Telehealth was used to support patients and carers, electronic record keeping and professional education. Notably, the number of home telemonitoring initiatives for patients had increased from the 2010 review. Despite this variety, many studies were small scale, descriptive and provided little evidence of evaluation of the service. Ten papers were sufficiently detailed to allow appraisal against the digital service standard and only one of these met all of the criteria to some extent. Seven studies made reference to emergency care access.

**Conclusions:**

Although there is growth of telehealth services, there remains a lack of evaluation and robust study design meaning conclusions regarding the clinical application of telehealth in palliative care cannot be drawn. There is insufficient evidence to appreciate any benefit of telehealth on access to emergency care. Future work is needed to evaluate the use of telehealth in palliative care and improve telehealth design in line with digital service standards.

## Background

Historically focussed on cancer care, there is a growing expectation that palliative care services should provide care to patients with any life limiting condition [1]. This, along with an ageing population with a growing burden of comorbidities has led to increasing strain on palliative care services. Several studies have shown the benefit of palliative care to patient quality of life [2, 3]. Despite this, work completed by Marie Curie highlights the inequalities in service provision for palliative care patients, with particular reference to patients with non-cancer diagnoses and out of hours care [1]. The provision of palliative care across rural communities should also be highlighted as a challenge for the specialist palliative care service [4].

Supporting patients with palliative care needs to access services and avoid hospital admission requires increasing support by community general and specialist palliative care services [5]. Several studies in the UK have indicated that the majority of patients wish to die at home [2], and a systematic review by Cochrane demonstrated that home-based end of life care increased the likelihood of dying at home [6].

Telehealth is a new and growing industry which comprises healthcare services, information technology and mobile technology. The World Health Organisation (WHO) state “Telehealth involves the use of telecommunications and virtual technology to deliver health care outside of traditional health-care facilities” [7]. The Health Resources and Service Administration expand on this description in their definition to include education; “the use of electronic information and telecommunications technologies to support and promote long-distance clinical health care, patient and professional health-related education, public health and health administration” [8].

The use of telehealth in a variety of chronic health conditions, such as heart failure and chronic obstructive pulmonary disease (COPD), has been studied [9, 10] and there is a growing body of research into its application in palliative medicine. Although there have been some large-scale notable uses of telehealth; for example, the use of electronic patient record systems in the UK [11], the importance of telehealth in providing quality healthcare has only recently become more widely accepted. Used to its full potential, telehealth technology could be particularly vital in improving access to healthcare over geographical distance and outside of normal working hours [12]. Telehealth has also been postulated as a solution to reduce acute hospital admissions which currently account for around 65% of hospital bed days in England [13].

The potential for telehealth to be utilised in the provision of palliative care services has already been identified in national publications. NHS Scotland identified ‘providing telehealth and telecare support’ as one of their actions as part of the ‘Living and Dying Well’ action plan [14] and the National Palliative Care and End of Life Care Partnership also identified the use of technology in their ‘ambitions’ for 2015–2020 [15]. In 2017, the UK government published its Digital Service Strategy which outline the ambitions to grow digital services across a variety of sectors [16]. This includes a service manual which explains how teams can build a good digital service. As a part of this work, the government also developed a Digital Service Standard [17]. This standard is a list of criteria to help create and run good digital services. The criteria include detailed information on why each element is important and what it means when designing and delivering a service. Some of the criteria include understanding user needs, making a service simple to use and protects user data. The digital service standard will be discussed further in the results section.

A review undertaken in the use of telehealth in palliative care in the UK was published in 2010 [18]. This paper examined the use of telehealth in palliative care settings in the UK between 1999 and 2009. The paper showed that telehealth was becoming increasingly accepted as useable by patients and healthcare professionals in this field; however there was an identified lack of clear evidence-based research to support the use of telehealth in palliative care in the UK [18].

Telehealth may provide a solution to meeting the growing demands of palliative care services across geographical regions with limited resources. Ready access to general and specialist palliative care services for patients with a variety of life-limiting conditions may prove beneficial in reducing the need for emergency services. This review will examine the current status of telehealth in palliative care in the UK and any evidence to suggest an effect on access to emergency or unscheduled care. In light of the recent UK government publications on digital services, the review will also examine to what extent current initiatives meet these standards. The issues described are not unique to the UK- studies in Australia [19, 20], the USA [21] and Europe [22] demonstrate a global interest in the potential of telehealth for meeting the needs of the palliative care population. Although this review focusses on UK studies, the results are undoubtedly transferable to an international audience.

## Methods

The protocol for this systematic review was registered with PROSPERO and can be found at http://www.crd.york.ac.uk/PROSPERO/display_record.php?ID=CRD42017080038.

The aim of this systematic review is to describe and evaluate the current use of telehealth technology in the provision of generalist and specialist palliative care in the UK. The authors examined the UK specifically in line with the 2010 review and also due to the unique way healthcare services- specifically, palliative care- are structured and funded via the National Health Service (NHS), meaning direct comparison with other countries would not be possible. The NHS is a government funded health and medical service which is free at the point of access to all UK residents [23]. Although a proportion of palliative care services are commissioned and funded by the NHS, much of palliative and end of life care is provided by hospices based in the voluntary sector [24]. To this end, the systematic review will answer the following question:

1. What are the current published uses of telehealth in palliative care in the UK?

In addition to the primary objective of describing telehealth use in palliative care, the review will also address the evaluation of these services. The secondary questions to be answered are:

2. If telehealth is being used for patients and/or carers, does this meet the criteria of a digital service as described by UK government?
3. Is there any evidence that compared with standard care, telehealth initiatives reduce the need for access to emergency/acute services for the palliative care population in the UK?

The authors used similar methods to those in the 2010 review paper in terms of selected electronic databases and grey literature searches. The search terms used for this review included those in the 2010 review however additional terms were added (adapted from previous Cochrane reviews [25, 26]) to maximise identification of suitable literature. Additionally, the authors opted not to include ‘United Kingdom’ and other synonyms in the search terms as it was felt this may result in missing relevant literature which referenced specific UK locations such as towns or regions.

Thematic synthesis was used to examine the results of the review [27].

### Inclusion criteria

Due to the descriptive nature of literature available, and initial scoping indicated the amount of literature available on this topic was not expected to be substantial, all study types including case series were included for review, with the exception of anecdotal opinion pieces and editorials. Research published on or after 1st January 2010 was included in this systematic review to allow comparison to the 2010 review article. Due to the nature of the review examining only studies from the UK, only articles reported in the English language were included.

Of interest were studies which described the use of any telehealth initiative in the delivery of palliative care in the UK. We included studies which described the use of telehealth to facilitate multi-disciplinary working or for the purpose of staff education and support, as well as to provide service to patients with palliative needs. Paediatric studies were included.

Studies describing the use of any mode of telehealth intervention were included, such as remote patient monitoring, digital support via telephone or video for patients or carers, remote support and advice for healthcare professionals in managing the stated population, or facilitation of the education and networking of healthcare professionals delivering palliative care.

### Outcomes

The primary outcome of the review was to describe telehealth use in palliative care in the UK. The secondary outcome was to judge whether the telehealth initiative described met the digital service standard. The UK government published its ‘Digital Service Standard’ in 2016 which is a set of criteria to help create and run good digital services [17]. The authors adapted these criteria and judged the telehealth interventions in the included studies against this standard. Additional outcomes included any reduction in the requirement of acute or emergency services when compared with standard care, cost effectiveness of telehealth interventions and participant perception of the technology.

### Search methods for identification of studies

We identified studies from a search of five databases conducted November 2017:

1. EMBASE
2. MEDLINE
3. CINAHL
4. Psychinfo
5. Cochrane central register of controlled trials

Search terms were adapted from existing strategies used in Cochrane systematic reviews [25, 26] and developed with input from our academic liaison librarian team (Table 1). Search terms were tailored to the five electronic databases accordingly.

**Table 1:** Terms used in search strategy

1. Identification of palliative care	
Palliative care OR Terminal care OR Terminally ill OR advanced/end stage/terminal disease/illness/cancer OR last year of life OR end of life OR macmillan/marie curie nurse	
2. Telehealth	
Telemedicine OR Telecommunications OR Telecare OR telemonitor OR teleconsult OR teleconference OR telephone OR telehealth OR remote consult OR ehealth OR mobile health	

A search of the grey literature with search terms modified from the above was also conducted using Google search engine. Additionally, we handsearched the conference literature from the European Association for Palliative Care, EThOS doctoral theses and reference lists from included papers. Where suitable abstracts were identified from conference or thesis abstracts, authors were contacted for full published papers and one expert in the field was approached for discussion. If full papers had not been published, these were excluded.

### Data collection and analysis

Results of the literature search were uploaded on to Covidence, an online tool to support literature screening. Two authors (SH and HJ) screened the titles and abstracts for relevance, to judge eligibility and remove duplicates. Full text of all potentially relevant studies was assessed by SH and HJ. Disagreements were resolved by consensus or by consulting a 3rd reviewer (AG or NP).

Data from each study were entered on a data extraction form. Specifically, data on the study population including sample size and diagnosis, study intervention, study design including data collection and analysis methods, and results or recommendations were extracted. The form was piloted by the two reviewers to ensure usability and consistency. All studies were extracted by SH with HJ independently completing data extraction on 80% of the studies included. Following extraction of baseline data (study type, number and nature of participants), thematic synthesis was conducted from the included studies following the methods of Thomas and Harden [27]. Following familiarisation with the study by repeat reading, any qualitative data was initially coded by hand and connections and overlaps in the data brought together in to descriptive themes. The themes were then reviewed in line with the review objectives to ensure the themes were able to capture crucial aspects of the data and address the review questions.

### Critical appraisal

Where possible, critical appraisal of the methodology of the paper was conducted using criteria adapted from Wallace et al’s 2004 paper on meeting the challenge of developing systematic reviewing in social policy [28]. A critical appraisal tool was not used in the 2010 review. This appraisal methodology allows for qualitative research evidence, which was felt to be important for this systematic review. The purpose of this appraisal was to provide an overview of the quality of the papers- studies were not excluded purely on the basis of the critical appraisal. Wallace et al’s original critical appraisal criteria can be found in Additional file 1.

## Results

**Fig. 1:**
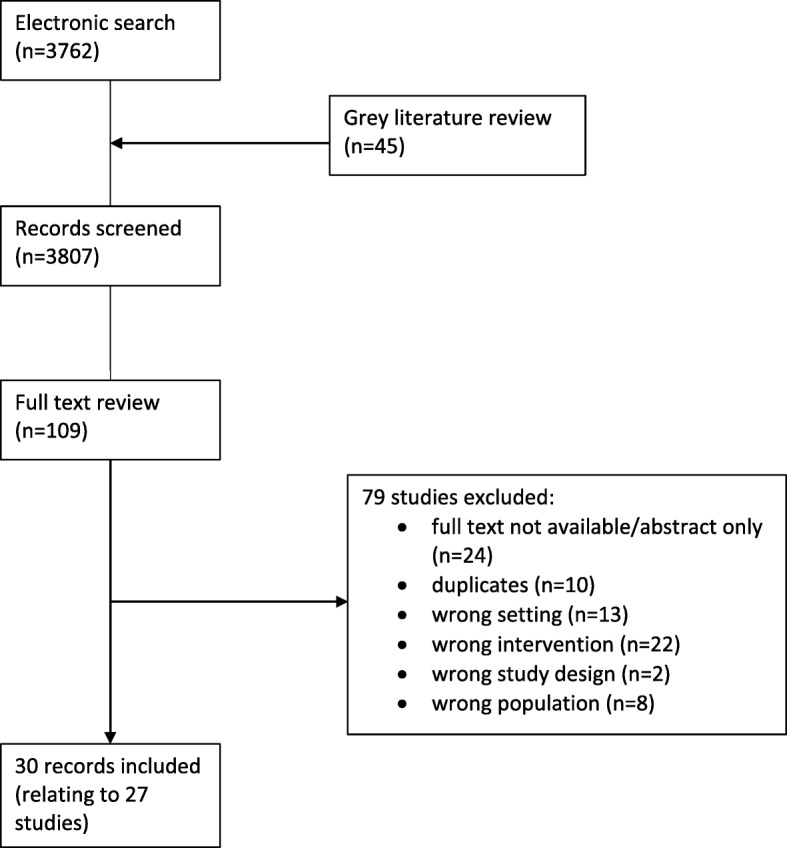
PRISMA flowchart of results

A total of 30 articles met the inclusion criteria and were included in the synthesis. The search results are shown as a PRISMA flowchart in Fig. 1.

A table of the included studies [22–49] can be found below [29, 30] (Table 2). Studies are grouped according to the predominant outcomes; quantitative, qualitative, protocols and mixed methods.

**Table 2:** Summary table of included studies

Author (year)	Research question	Participants	Telehealth initiative	Setting	Key findings	Overview of quality
Quantitative						
Chatwin (2016) [22] Randomised crossover trial	Effect of home telemonitoring on interaction with acute services and quality of life and self-efficacy	68 patients with COPD or non-COPD respiratory failure	Philips Motiva home telemonitoring system with heart rate monitor, pulse oximeter, blood pressure monitor and weighing scales	Patient’s home	Admission rates and home visits increased in the telemonitoring arm Time to first exacerbation did not differ between groups. Self-efficacy fell in the telemonitored group.	**Score = 18**
Dey (2016) [25] Prospective interventional pilot study	Acceptability of technology to monitor symptoms in advanced peritoneal dialysis patients	22 adults with end stage renal failure on peritoneal dialysis	Specialised software on tablets for inputting symptom data and blood pressure measurement	Patient’s home	Claimed 36 admissions avoided, support to manage at home provided on 154 instances. 91% retention rate in study excluding medical reasons. No significant change in QOL scores. QUEST scores high indicating satisfaction with the technology.	Score = 12
Dierckx (2015) [26] Retrospective observational analysis	Does home telemonitoring reduce heart failure mortality?	333 patients with heart failure referred- 278 agreed to telemonitoring	Motiva home telemonitoring system for symptom data and clinical measurements. Can also transmit educational videos.	Patient’s home	Telemonitoring was associated with reduced all-cause mortality and overall improved survival. The number of admissions and time to first hospitalization was the same in each group. Patients who refused telemonitoring were generally older.	Score = 10
Hall (2013) [29] Service evaluation	An evaluation of the 7 day specialist palliative care service	N/A	Telephone advice service within an acute hospital	Hospital	99 calls with professionals and 37 with carers in a one-year period. Consultant contacted on average once per day at the weekend.	N/A
Hamad (2015) [31] Prospective observational	Description of the use of telehealth data in a COPD MDT	95 patients	Telehealth platform via smartphone or tablet to provide symptom status and oxygen saturations	Hospital	18 of 95 patients had no recommendations from the MDT. There were 141 recommendations generated for the remainder eg. referral to palliative care, smoking cessation.	N/A
Lewis (2010) [32] Randomised controlled trial	Does home telemonitoring reduce healthcare use in COPD?	40 patients with COPD	HealthHub system via Freephone line to monitor symptoms and pulse oximetry.	Patient’s home	Reduced contact with primary care in intervention group- not statistically significant but may be clinically important. No difference in ED attendance or hospital admissions. Patients uploaded median 97% data and no difficulties using technology.	**Score = 17**
Plummer (2011) [33] Service evaluation	Analysis of the specialist palliative care advice line	70 patients and carers	24/7 telephone advice line for patients, carers or professionals	Hospice	Most calls weekday after 5 pm and from patients or carers. Primary reason for call was symptom management. 65% callers remained at home following call.	Score = 8
Purdy (2015) [34] Retrospective observational study	Impact of the delivering choice programme on place of death and hospital usage	Analysis of 3594 patients	Electronic end of life register, out of hours advice line (plus two non-telehealth components)	Hospice and community	21–24% accessed some element of the programme. Patients using programme more likely to have cancer diagnosis. Care coordination centres most effective intervention. OOH advice line associated with reduced ED attendance in last week of life only. Patients using centres or entered on end of life register were less likely to die in hospital. Hospital admissions were lower in both counties for patients using the programme in 30 days prior to death.	**Score = 18**
Warren (2011) [35] Prospective observational study	Review of telephone support for patients with advanced breast cancer	229 calls related to patients with metastatic breast cancer	Telephone advice line	Hospital	Largest contact group was patients followed by professionals. Total time spent on calls was 63 h (30% of CNS working time). 1281 interventions generated from the calls.	Score = 12
Wye (2016) [36] Service evaluation	Analysis of electronic palliative care coordinating systems (EPaCCS)	101 health care professionals	Electronic palliative care record	Community	EPaCCS used in small proportion of patients (9–13%). Where EPaCCS used, seems to correlate with a home death.	**Score = 17**
Qualitative						
Carlebach (2010) [21] Qualitative interviews	What are the experiences and opinions of users of telephone support service?	Palliative care diagnosis not specified. 6 patients, 8 carers, 13 health professionals	Hospice 24 h advice line	Hospice	Relatives and carers were group most likely to use service, followed by district nurses. The service was valued by users.	Score = 8
Duxbury (2015) [27] Qualitative interviews	What are the barriers and enablers to adoption of Coordinate My Care?	8 professionals	An online tool completed by health professionals for palliative patients	Community	Useful and relevant however process of completing ‘laborious’ and issues with connectivity- lack of remote access and some providers not connected.	N/A
Faull (2016) [28] Description of initiative	Description of online learning tool.	For professionals	e-ELCA online education tool for palliative care professionals	N/A	None- description only.	N/A
Hall (2012) [29] Qualitative interviews	The opinion of electronic palliative care summary record in Scotland	16 professionals, 6 patients/carers	Electronic palliative care system to allow recording of patient data and sharing with out of hours services	Community	Useful and feasible innovation. Felt to be more specific to cancer patients. Felt that not enough emergency providers know of its existence. Reassuring for patients and carers.	Score = 11
Hobson (2018) [37] Qualitative interviews	Identify how technology may improve the service for motor neurone disease	3 patients, six carers and 1 motor neurone disease specialist nurse	Tablet computer app which patients use to input health and wellbeing data	Hospice/ Community	Telehealth was acceptable to patients and carers. They wanted more information to help self-manage. The touch screen layout will be redesigned following observation of users.	Score = 15
Johnston (2011) [38] Qualitative interviews	Evaluating the use of telehealth in palliative care across Scotland	22 patients and carers, 8 healthcare professionals	Variety of telehealth interventions discussed	Hospice/ Community	Patients generally unaware of the term ‘telehealth’ but aware of the existence of technologies. Stakeholder telehealth activity consisted of videoconferencing for MDT, networking and education. Patients/carers aware of telephone advice lines, internet forums and personal safety alarms and found these useful. Felt to be used more in remote locations. Felt should supplement rather than replace existing support. Barriers to use were broadband coverage, funding and lack of awareness.	Score = 14
Leadbeater (2014) [39] Qualitative interviews	To review the organisation of community palliative care teams in rural England	6 specialist palliative care nurses	Variety of telehealth interventions discussed	Community	Two teams used videoconferencing for MDT meetings. 40–75% patient contact via telephone. 3 teams had laptops and 2 had remote access to patient records.	N/A
Middleton-green (2016) [40] Qualitative interviews	Evaluation of palliative care telephone advice line	8 patients and 6 carers	Telephone advice line or video call from iPad with hub staffed by nursing professionals	Hospice	5106 telephone calls received related to 1813 patients. Service found to be beneficial for emotional support and practical advice. Reports from patients of how advice prevented avoidable admission or expedited appropriate admission. Reported 98.5% of calls resulted in patients remaining in place of residence	Score = 10
Nwosu (2012) [41] Description	Describe smartphone applications for palliative medicine	N/A	Smartphone applications	N/A	Six applications identified- 2 ‘blog’ style, 2 with guidance to facilitate learning or practice and 2 apps to facilitate opioid prescribing.	N/A
Rafter (2016) [42] Description of service	Description of e-health in managing pressure ulcers in palliative care	2 case examples	e-Health system for staff to upload information/picture of ulcers.	Hospice	All patients received care pathways within 24 h of referral. Staff gave positive feedback- easy to use and increased job satisfaction.	N/A
Wye (2014) [43] Realist evaluation	‘Real life’ evaluation of what facilitates home deaths and reduces hospital admissions for palliative patients	43 family carers and 105 professionals	Electronic end of life register, out of hours advice line (plus two non-telehealth components)	Hospice/ Community	Having skilled, experienced professionals with sufficient and dedicated time was important to the success of the project. Overall there was high carer satisfaction, low hospital utilization and more deaths in the community among project users. Patchy uptake of the service.	**Score = 17**
Protocols						
Aiyegbusi (2017) [20] Study protocol	Does the use of patient-reported outcome measures promote care and safety in managing advanced CKD?	Stage 4 or 5 chronic kidney disease.	Electronic questionnaire on smartphone/ tablet/laptop/ computer of symptoms	Patient’s home	N/A	N/A
Choyce (2017) [23] Study protocol	Effect of home telemonitoring on clinical parameters and quality of life	Cystic Fibrosis patients with admission for IV antibiotics in last 24 months	Mobile phone for symptom reporting and Bluetooth spirometer	Patient’s home	N/A	N/A
Hudson (2016) [44] Study protocol	Feasibility of online cognitive behavioural therapy intervention	End stage renal failure on dialysis with depression or anxiety	Online cognitive behavioural therapy using an iPad accompanied by telephone support.	Patient’s home	N/A	N/A
Mixed						
Cox (2011) [24] Intended mixed-methods. Qualitative interviews with clinicians	To assess the acceptability of technology to monitor symptoms following palliative radiotherapy for lung cancer	Patients with lung cancer receiving radiotherapy- none successfully recruited	CareHub device to monitor symptoms reported by patients	Patient’s home	21 patients identified, consent from clinician withheld for 20. 1 other patient declined to participate. 9 of 13 clinicians felt e-technology inappropriate in this group due to age. Themes of gatekeeping due to concern of burden of research on this population. Concerns their clinical judgement replaced by technology.	Score = 9
Hattink (2015) [45] Randomised controlled trial	Does an e-learning course increase empathy and understanding in dementia caregivers?	57 caregivers of people with advanced dementia	Web-based training portal on different aspects of dementia care	Carer’s home	30/57 UK participants did not complete the course. Modules rated useful and user-friendly. Empathy, perspective and coping with stress improved in the intervention group (though UK/Dutch results pooled).	**Score = 16**
Hudson (2017) [46] Randomised controlled trial	Feasibility of online cognitive behavioural therapy (CBT) intervention	25 patients- 18 intervention, 7 control	Online cognitive behavioural therapy using an iPad accompanied by telephone support.	Patient’s home	410 patients approached and 25 agreed to participate with 23 completing follow up. Adherence with online CBT higher in control arm. Numbers not large enough to show statistical difference for patient reported outcomes. Calculated QALY gain for supported arm £82,283 though wide confidence intervals.	**Score = 17**
Lisk (2012) [47] Mixed methods	Does geriatrician input for nursing home patients reduce emergency admissions?	Audit of 1954 nursing home residents with 3 nursing homes involved in pilot	Telephone advice line to speak to geriatrician and e-mail alert to geriatrician when patient admitted	Hospital and community	Reduction of bed days from 90 to 33 in initial pilot with calculated cost saving of £2630. In second phase of study calculated reduction of 250 bed days over 4 months with potential cost savings of £74,383. Service was well received by GPs.	N/A
Milton (2012) [48] Service evaluation	Describe the 7 day community specialist palliative care service	20 patients/carers 6 professionals	Proactive and reactive telephone support run by community specialist nurses	Community	36% of contact from the service was unplanned. There were 132 telephone contacts in a 6 month period. Viewed positively by all staff in the focus group and valued by patients.	Score = 9
White (2016) [49] Prospective longitudinal cohort study	Review of ‘ECHO’ education project for hospice nursing staff	34 community hospice nurses	Weekly educational session facilitated by videoconferencing	Hospice	28/34 completed pre and post intervention evaluation. Mean knowledge score improved by 11.3% (*p* = 0.0005) and all domains of self-efficacy improved. Project received positively by participants.	**Score = 18**

Studies with overview of quality scores highlighted in bold in the table met all of the nine quality criteria completely or to some extent

### Overview of studies

A wide variety of study designs were used, with the most common being qualitative methods used in seven of the papers [32, 35, 36, 38, 41, 42, 50]. Four of the papers were service evaluations [30, 47, 51, 52] and there were three randomised controlled trials [34, 48, 49]. Three of the papers were protocols [29, 33, 45] and three of the papers simply gave a description of the intervention without any identifiable study design [39, 53, 54] which served to address objective 1 of the study (description of current telehealth use). Other study designs included randomised crossover trial [31] mixed methods [37, 43] realist evaluation [55], prospective interventional [44], prospective longitudinal cohort [56], prospective observational [40, 57] and retrospective observational [46, 58].

The majority of included studies had relatively small sample sizes. Where qualitative or service evaluation type studies were conducted that involved interviews with participants, the majority had sample sizes less than 30 which may be expected given the stated methodology. The exception to this is in the two studies conducted by Wye et al. [30, 55] where 148 and 101 participants were interviewed respectively. Similarly, in the studies which examined an intervention, sample sizes were low (range 22–68) and the median number of participants was 40.

Fourteen of the included studies had a mix of participants (patients, carers and professionals) [30, 32, 36, 37, 40–43, 47, 50–52, 55, 57], nine were patient centred [29, 31, 33, 34, 44–46, 49, 58]and five were studies with telehealth interventions aimed at professionals [35, 38, 39, 54, 56]. Only one study by Hattink et al. [48] described a telehealth intervention specifically for carers; an online education tool for carer-givers of those with advanced dementia. One study was a description of palliative care mobile phone applications and did not have any participants [53].

Where studies included patients, the majority did not specify a particular diagnosis [32, 34, 36, 42, 43, 47, 50–52, 54, 55, 58]. Specific diagnoses included end stage renal disease, COPD, cystic fibrosis, heart failure, dementia, motor neurone disease and specific cancer sites (lung and breast).

### Overview of quality

Using guidance provided by Wallace et al’s 2004 paper [28], the authors appraised the methodology of 19 of the 30 papers. For the 11 which were not able to be assessed, this was due to the paper being descriptive in nature with insufficient detail on study design.

The 19 papers suitable for appraisal were reviewed against a set of nine criteria developed by the authors which had been adapted from Wallace et al. [28] Two authors independently scored eligible papers against the nine criteria. Authors judged whether papers met the criteria fully (scoring 2), to some extent (scoring 1), not at all or unable to say from the information in the paper (scoring 0). The maximum score was therefore 18; scores for suitable papers are included in the table below.

Eight of the 19 papers met all of the nine criteria completely or to some extent [30, 31, 34, 48, 49, 55, 56, 58]. These papers scores are highlighted in bold in the table. The majority of papers which did not meet the nine criteria did so because of insufficient sample to explore the subject, or insufficient description of data collection methods.

### Types of telehealth interventions

The first objective of this review was to describe the current uses of telehealth in palliative care in the UK which will be discussed here.

There was a variety of telehealth interventions described in the included studies. The majority of interventions described a form of home telemonitoring (using telephone or computer software to record clinical symptoms or signs from the patient’s home) [29, 31, 37, 40, 41, 44–46, 49]. Home telemonitoring was used in a variety of conditions; respiratory disease, heart failure, motor neurone disease, cystic fibrosis and end stage renal failure. This required patients to input data using their telephone landline, their television or using computer hardware and software provided to the patient for this purpose. All home telemonitoring studies required patients to input specific data regarding symptoms specific to their illness, such as breathlessness in respiratory disease, and some studies also required patients to provide physical parameters. For example, pulse oximeter measurements in respiratory disease studies [31, 49] and weight and blood pressure measurements in heart failure and renal failure studies [44, 46]. The majority of these studies included some form of telephone support either in response to patient data triggering an alert or as an additional support for patients at home.

Several papers described a telephone advice line as the telehealth intervention [36, 47, 50–52, 57]. These were a mix of ‘reactive’ and ‘proactive’ telephone services and tended to be for the palliative population generally rather than a specific diagnosis. The majority of these papers were descriptive of the service and used qualitative measures to obtain feedback from users on the service.

Three studies described the use of electronic patient records as a telehealth intervention [30, 32, 38]. Five studies described the use of online or videoconferencing platforms to facilitate education; these were either to support patients and carers [33, 34, 48] or to provide education and networking opportunities for professionals [39, 56].

A number of studies had a mix of interventions- from studies evaluating participant opinion on a number of interventions [35, 42], to studies which had a combination of elements to their intervention (a combination of telephone advice line, electronic patient record and non-telehealth interventions such as ‘end of life care facilitators’) [43, 55, 58].

### Synthesis of findings

Results from included studies have been grouped in to quantitative and qualitative outcomes. Quantitative results included descriptive data on the number and demographic information of users of a telemedicine service. Studies which examined specific outcomes such as number of acute hospital admission, length of admission, primary care contacts and number of contacts needed from a telemedicine provider tended to be associated with studies which described use of a home telemonitoring system. Some quantitative data was specific to the condition monitored, for example spirometry values in cystic fibrosis patients. The study by Lisk et al. which described a multi-modal intervention of telephone advice line, multi-disciplinary team meetings and e-mail alerts on hospital admission for nursing home patients discussed cost reduction as a result of the intervention [43]. The study calculated a saving of £2630 as a result of their intervention and predicted a £74,383 cost reduction for the next, larger-scale stage of their study. These calculations were reached by comparing the number of inpatient bed-days during the intervention period with the same period from the previous year and hence are estimations. It is not possible to ascertain which element of their intervention resulted in this outcome.

Qualitative results included participant and healthcare provider opinions on either a specific service or telemedicine in general. The results from these studies were generally positive and indicated an openness to telemedicine in palliative care [32, 36, 38, 41, 42].Specifically, healthcare professionals reported telemedicine interventions to be ‘useful’ and ‘relevant’ whilst patients reported telehealth as an acceptable provision of care [32, 38, 41].However, the study by Johnston et al. [42] also highlighted some of the potential barriers to telehealth following interviews with patients, carers and healthcare professionals, including a need to improve infrastructure to support telehealth, perceived potential for patient difficulty in managing the technology and a lack of funding and broadband coverage. The paper by Cox et al. [37] aimed to introduce a home telemonitoring system for patients receiving palliative radiotherapy in lung cancer. Unfortunately, the study was unable to take place due to refusal of consent to approach patients by their clinicians. On interviewing clinicians regarding this there was clear evidence of gatekeeping preventing participation in the research; many clinicians felt technology was inappropriate in this specific population due to age, burden of illness and rapidity of deterioration.

A number of the studies did not outline specific outcomes and were purely descriptive of the telemedicine service or intervention.

### Telehealth and the digital service standard

The second objective of this review was to assess whether telehealth initiatives met the standard of a UK digital service set out by the UK government; this will be discussed here.

Ten papers from the review were suitable for review of the telehealth intervention using criteria adapted from the UK government’s digital service standard [17]. The remaining 20 studies did not contain sufficient detail of the service to complete this review, or detailed a multi-faceted intervention where telehealth was only a component.

Two authors (SH and HJ) independently scored eligible papers against eight criteria using the same system as above (criteria met fully, to some extent, not at all or unable to say). Of these 10 papers, only one paper met all 8 criteria completely or to some extent (Table 3) [44].

**Table 3:**
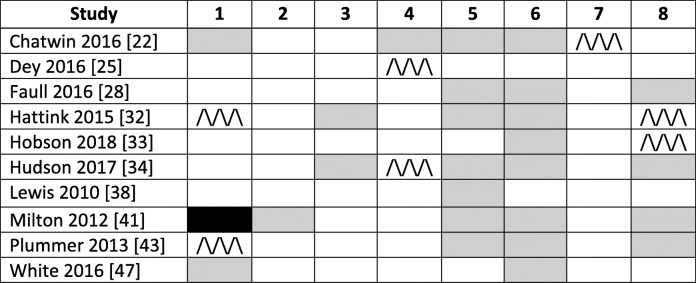
Performance of studies suitable for telehealth intervention appraisal, where white is ‘meets all criteria’, /\ pattern ‘to some extent’, black is ‘not at all’ and grey is ‘unable to say’

It is clear from the table above that the majority of papers did not detail any information on security and privacy, and making a plan for being offline. Identification of performance indicators and collecting performance data was also not detailed or only partially detailed by many of the included papers.

### Access to emergency care

The third objective of this review was to examine for any evidence of reduction in access to emergency care as a result of the telehealth initiative; this will be discussed here.

Seven of the studies made specific reference to reduction in access to emergency or acute care services [31, 36, 44, 46, 49, 52, 58].

Two of the studies which examined the use of telephone advice lines reported a reduction in admission. In the Middleton-Green et al. study [36], the authors state that ‘98.5% of calls resulted in patients remaining in their place of residence’ and the service evaluation of the telephone advice line carried out by Plummer et al. [52] discusses that 70% of callers were not admitted to hospital- possibly as a result of advice given by the call handler.

The studies by Wye et al. [55] and Purdy et al. [58] describe the ‘Delivering Choice Programme’ (DCP). In their study, Purdy et al. describe how participants receiving DCP were less likely to die in hospital, less likely to be admitted to hospital in the 30 days prior to death and less likely to visit the emergency department.

Four studies of home telemonitoring discussed access to emergency care. Dey et al. [44] found that 36 admissions were avoided using their home telemonitoring system in renal failure patients. Dierckx et al. [46] undertook a retrospective observational analysis of telemonitoring in heart failure patients and described that telemonitoring seemed to be associated with a reduction in all-cause mortality, however the number of admissions due to heart failure and the time to first hospitalisation was similar between the two groups (receiving telemonitoring and not). The randomised crossover trial by Chatwin et al. [31] examining telemonitoring in respiratory failure resulted in increased respiratory admissions and home visits in the telemonitoring group. Interestingly, the randomised controlled trial of telemonitoring in COPD patients by Lewis et al. [49] showed no difference in hospital admissions, days in hospital or emergency department attendances, but a reduction of contact with primary care though this was not statistically significant as the study was underpowered.

## Discussion

Similar to the review published by Kidd et al. in 2010 [18], this paper provides a useful overview and description of how telehealth initiatives are being used in palliative care in the UK. It is notable that despite a growth in the use of technology, the numbers of papers eligible for inclusion have not increased as expected. During the search, the authors noted a lack of conversion of abstracts to full publication; 12 interesting and potentially eligible abstracts were identified during the grey literature search which had not been converted to full publication. In keeping with these observations, Hanchanale et al. report that just over half of palliative care conference abstracts subsequently go on to full publication [59]. Although a Cochrane review in 2007 demonstrated a similar publication rate across all specialties [60], the article by Walshe in 2017 highlights the trend for observational rather than interventional research and a low publication rate of trials in palliative care [61]. This may account for the relatively low numbers of studies.

Despite this, we have found a number of papers which describe the varying uses of telehealth. Although telephone advice lines and the use of telehealth in providing patient or professional education continue to feature in this review, there was a notable increase in the number of home telemonitoring initiatives implemented compared to the 2010 review. This may be due to the improvement in these technologies. It was interesting to note that all of the home telemonitoring studies were undertaken in participants with specific diagnoses (for example, heart failure) rather than a general palliative care population. This may be appropriate given different diagnoses may result in different symptoms but may also be a barrier when thinking about the number and variety of telehealth applications needed to meet the demands of the palliative care population as a whole.

Where telehealth is described, the detail included in the paper was often insufficient for the authors to judge the initiative against the digital service standard. The majority of the papers which could be judged against this standard did not meet the criteria. This may reflect how recent this digital service standard was published (some papers included were published prior to the standard) but may also corroborate with the overall lack of robust study design noted across the review. Given this standard is now widely available, it may be that future telehealth initiatives align with the criteria more closely. It is worth noting that the criteria were adapted by the authors. For example, the requirement to ‘test with the minister’ was felt to be inappropriate for this review. The digital service standard was updated in July 2019 after completion of this review and the new criteria seem to reflect some of the challenges identified, including removal of the aforementioned point [62].

Kidd et al. [18] comment that telehealth is reported to advantage patient care by improving the patient and carer experience, however there is little known about the clinical benefits and feasibility of providing telehealth services. This review includes papers which comment on clinical benefits and reduced need for emergency care but there are limitations to these findings. Purdy et al. find a reduction in hospital admission, emergency department attendances and deaths in hospital, however their intervention was multi-faceted and they acknowledge that their ‘coordination centres’ which organise care and equipment for patients seemed to provide the most benefit, rather than the telehealth aspects. Although Dey et al. [44] report that admissions were avoided, the sample size for the study was small and it is unclear how the authors have reached this conclusion. Dierckx et al. [46] report a reduction in all-cause mortality, however this was a retrospective observational study where patients were offered telemonitoring rather than allocated. If patients opting to engage with telemonitoring are in general more engaged with healthcare, this may account for some of their findings. The study design in the paper by Middleton-Green [40] was insufficient to demonstrate that patients remaining in their usual place of residence was as a direct result of their telehealth initiative.

Although there is an increase in the use of home telemonitoring, and a growing appreciation for the use of telehealth in palliative care (as evidenced by the qualitative nature of some of these studies) there remains a lack of evaluation of telehealth interventions. Where evaluation was undertaken, it was difficult to attribute the results to telehealth as many studies implemented a multi-faceted intervention (for example, telephone advice line with a face-to-face support element). Most of the literature continues to be purely descriptive and without robust study design. Without this, it is not possible to clearly demonstrate a clinical benefit of telehealth in palliative care in this review.

### Limitations

There are several limitations to this review. Although article screening and data extraction was conducted by two reviewers, the synthesis was conducted by only one reviewer which limits the objectiveness and introduces opportunity for error. The studies were not homogenous in nature, which also makes synthesis of the findings difficult. The variety of key terms used in the literature for both palliative care and telehealth made searching for articles challenging and although the search was thorough, omission of relevant articles cannot be ruled out.

It is worth noting that although the criteria used for review of study quality were adapted from existing literature, they were developed by the authors and assessed by the authors, creating scope for bias. Rather than being used as a specific and rigorous assessment of each paper, it served to emphasise the lack of clear study design or method used in the majority of the included papers, resulting in many of these studies being very difficult to reproduce. It is also worth noting that the failure of some papers to meet the nine criteria may actually reflect the written report of the study, rather than the rigor of the method. Similarly, the criteria used by the authors to judge telehealth initiatives against the digital service standard are subject to similar levels of bias for the reasons detailed above.

### Strengths

Despite these limitations, the included studies and synthesis have been able to address the three review questions. The literature search was conducted rigorously and is replicable. Inclusion of all applicable studies in the review allowed for a broad overview of this topic. Screening of papers, data extraction and assessment of quality were undertaken by two reviewers to attempt to minimize bias. Interpretation and synthesis of themes was discussed amongst all authors. The results reinforce some of the findings from the 2010 review used as a starting point for this review and go further to examine some new areas relevant for future work, such as the comparison against the digital service standard.

### Impact on policy and practice

Although confirming that telehealth initiatives continue to be implemented in palliative care, this review highlights the need for further studies on the use of telehealth in palliative care. Important questions regarding the acceptability and effectiveness of telehealth in this setting remain unanswered.

It was also noted by the authors that although some included studies examined the relationship between telehealth and access to emergency care, none of the studies specifically examined the effect on out of hours service provision. The palliative care and end of life priority setting partnership, identified the provision of palliative care outside of normal working hours as it’s number one priority [63]. This, coupled with the service delivery guideline for adults in the last year of life currently in progress by NICE [64] indicate that planning of specialist palliative care service provision is of great importance. Hence, the authors suggest that future work examining the use of telehealth in meeting the demands of an out of hours specialist palliative care service could have significant impact on future clinical practice.

## Conclusions

This review demonstrates that a variety of UK palliative care telehealth initiatives continue to be described in the published literature. Since the 2010 review there particularly appears to have been an increase in the number of home telemonitoring interventions, perhaps because of an improvement in this technology. However, where sufficient detail of the telehealth initiative allowed review against a standard, the majority of interventions did not meet the requirements of a UK digital service. Despite the description of telehealth development and implementation, there remains a lack of robust study design and evaluation of these interventions meaning that clear conclusions around the benefit of telehealth in palliative care cannot be drawn; there is insufficient high quality evidence to comment on any influence on access to emergency or unscheduled care. Further work to evaluate the use of telehealth in palliative care, and to specifically examine its use in out of hours specialist palliative care provision is recommended.

## Supplementary information

**Additional file 1.** Critical appraisal criteria from Wallace et al. (2004) [19].

## Data Availability

No primary data collected in this study. Detailed search strategy and further information on included studies available on request from Dr. Sophie Hancock.

## Abbreviations

CBT: Cognitive behavioural therapy
CNS: Clinical Nurse Specialist
COPD: Chronic Obstructive Pulmonary Disease
DCP: Delivering Choices Programme
ED: Emergency department
EPaCCS: Electronic Palliative Care Coordination System
MDT: Multi-disciplinary team
NHS: National Health Service
NICE: The National Institute for Health and Care Excellence
OOH: Out of hours
QALY: Quality adjusted life years
QOL: Quality of life
UK: United Kingdom
USA: United States of America
WHO: World Health Organization
