# Supplementary figures and images for: Telehealth in palliative care is being described but not evaluated: a systematic review
Source: BMC Palliat Care. 2019 Dec 13;18:114. doi: 10.1186/s12904-019-0495-5 (PMC6911274; doi:10.1186/s12904-019-0495-5)

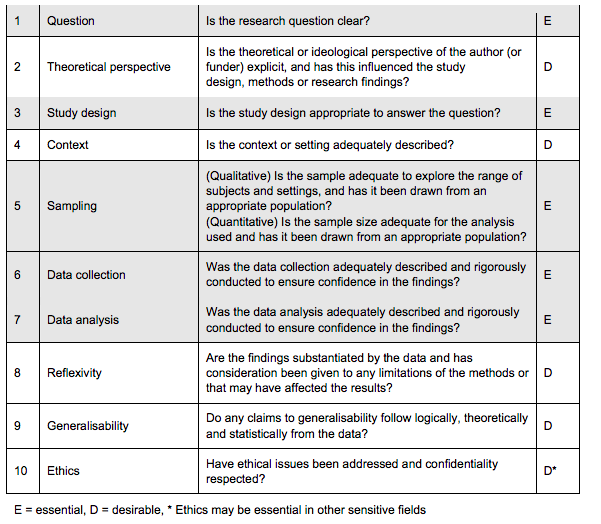
Additional file 1: Critical appraisal criteria from Wallace et al (2004).[19]

Supplement: Supplementary file 1 — Additional file 1. Critical appraisal criteria from Wallace et al. (2004) [19]. [file 12904_2019_495_MOESM1_ESM.docx]
